# Supplementary material for: The prevalence of AstraZeneca COVID-19 vaccine side effects among Nigist Eleni Mohammed memorial comprehensive specialized hospital health workers. Cross sectional survey
Source: PLoS One. 2022 Jun 24;17(6):e0265140. doi: 10.1371/journal.pone.0265140 (PMC9232121; doi:10.1371/journal.pone.0265140)
Supplement: Attachment — Submitted filename: Response to Reviewers.docx [file pone.0265140.s002.docx]

**Rebuttal Letter**

We thank the editor and the two reviewers for their comments on our manuscript. Below is our response to each point raised by the academic editor and reviews. We hope that we satisfyingly addressed them and the manuscript will be now suited for publication.

Sincerely,

*Corresponding author

Gelana Garoma

On behalf of all authors

Departemnt of Maxillofacial, Addis Ababa University, College of health sciences, School of medicine, Addis Ababa, Ethiopia

Email: [gelana.garoma2021@gmail.com](mailto:gelana.garoma2021@gmail.com) (GG)

**Academic editor**

1. File naming was edited to comply with the style requirements. We hopefully have no divergences from the style requirements now.
2. The authors received no specific funding for this work.
3. ORCID Id for corresponding author in Editorial manger included
4. Author Ajay Prakesh included
5. reference list reviewed
6. Language, spelling and grammar was checked online http://Prepostseo.com

**Reviewer #1**

1. Covid 19 status of our participants inquired and the finding included in result part after the reviewer comment made
2. Concurring comment about one figure about prospective of the study, we made the summary in conclusion part by sentences. If we miss understand it , please more clarification and ready to correct it.
3. English of manuscript was polished for publication
4. the Abbreviation was cross validated and defined in first use
5. Discussion was made for symptoms following COVID 19 vaccine

**Reviewer #2**

1. English was polished
2. Few things added about Covid 19 in Ethiopia and the available vaccine in Ethiopia during our study period was only AstraZeneca. So that comparative study was not done
3. We tried to correct formatting errors in manuscript and reference.
4. Challenges of this study separately discussed under limitation of the study
